# Linear Immunoglobulin a Bullous Dermatosis in Children

**DOI:** 10.3389/fped.2022.937528

**Published:** 2022-07-08

**Authors:** Francesca Mori, Francesca Saretta, Lucia Liotti, Mattia Giovannini, Riccardo Castagnoli, Stefania Arasi, Simona Barni, Carla Mastrorilli, Luca Pecoraro, Lucia Caminiti, Gian Luigi Marseglia, Annick Barbaud, Elio Novembre

**Affiliations:** ^1^Allergy Unit, Department of Pediatrics, Meyer Children's University Hospital, Florence, Italy; ^2^Pediatric Department, Latisana-Palmanova Hospital, Azienda Sanitaria Universitaria Friuli Centrale, Udine, Italy; ^3^Department of Pediatrics, Salesi Children's Hospital, AOU Ospedali Riuniti Ancona, Ancona, Italy; ^4^Pediatric Clinic, Department of Pediatrics, Fondazione IRCCS Policlinico San Matteo, University of Pavia, Pavia, Italy; ^5^Translational Research in Pediatric Specialties Area, Division of Allergy, Bambino Gesù Children's Hospital (IRCCS), Rome, Italy; ^6^Pediatric Unit and Emergency, University Hospital Consortium Corporation Polyclinic of Bari, Pediatric Hospital Giovanni XXIII, Bari, Italy; ^7^Pediatric Unit, Department of Surgical Sciences, Dentistry, Gynecology and Pediatrics, University of Verona, Verona, Italy; ^8^Department of Human Pathology in Adult and Development Age “Gaetano Barresi”, Allergy Unit, Department of Pediatrics, AOU Policlinico Gaetano Martino, Messina, Italy; ^9^Sorbonne Universités, Service de Dermatologie et d'Allergologie, Hôpital Tenon, Paris HUEP, APHP, Paris, France

**Keywords:** linear IgA bullous dermatosis (LABD), newborn, children, drug hypersensitivity, epidemiology, diagnosis, treatment

## Abstract

Linear Immunoglobulin A Bullous Disease (LABD) is a rare dermatosis whose pathomechanisms are not yet completely understood. LABD has different features characterizing adults and children in terms of potential triggers, clinical manifestations, and prognosis. The aim of the present study is to review all neonatal and pediatric cases of LABD and summarize the major characteristics. Childhood LABD is mainly idiopathic with a benign prognosis. Neonatal cases are difficult to differentiate from infectious diseases and usually have a poor prognosis. Drugs are one of the possible triggers that can activate autoimmune responses through antigen mimicry and epitope spreading as well as different stimuli (e.g., infections, inflammatory diseases, trauma). The gold standard for the diagnosis is based on direct immunofluorescence. Prognosis is generally favorable but often depends on the prompt dermatological diagnosis, treatment and follow-up guaranteed by a multidisciplinary team, including pediatricians for this group of age.

## Introduction

Linear Immunoglobulin A Bullous Disease (LABD) is a rare cutaneous disease characterized by subepidermal linear deposition of immunoglobulin A (IgA) along the basement membrane zone. LABD was first described in the late 1970s as Chronic Bullous Dermatosis of Childhood (1).

The two forms of the disease, one found in adults and one in children, have different clinical features but share the same immune-pathogenesis and microscopic findings (2). In children, it is more frequently idiopathic, but some drug-induced cases have been reported (3–8). Concomitant infections or malignancy are more often reported in adults, but their role has not yet been clearly defined. The pediatric form has a peak onset age of 4.5 years old (2), although it may also affect newborns, with a worse prognosis (9–13).

## Epidemiology

Incidence is estimated to be 0.2–2.3 cases per million/year (14), and it seems to be higher in South Africa, North Africa, and Asia (15–18). So far, no gender or ethnicity prevalence has been observed. A PubMed research has been performed using the specific research strings, retrieving a total of 145 items for “linear IgA bullous dermatosis, humans, English, child: birth−18 years, 1976–2022” and 169 items for “linear IgA bullous disease, humans, English, child: birth−18 years, 1976–2022.” Most of these are case reports or small case series. Despite this lack of knowledge, LABD is the most frequent chronic autoimmune blistering disease in children (10, 19–22).

### Pediatric Case Series

Spontaneous or drug-induced pediatric case series with complete clinical data are summarized in Tables 1, 2. Among a case series of 54 patients with the autoimmune bullous disease, there were 25 children (with a mean age of 4.5 years) who had a chronic bullous disease during childhood (2). Wojnarowska et al. (2) emphasized that 38% of children in this study had a previous infection (in the upper respiratory tract), and 31% of all patients were exposed to a drug (these were mainly anti-inflammatory drugs; these data were not reported for the pediatric subgroup). Some other case reports have been published reporting toddlers or young children with LABD mainly induced by antibiotics (3–8). Since vancomycin is not frequently used among children, it is more frequently reported as a causative drug in adults.

**Table 1:** Pediatric cases of LABD: epidemiological and clinical findings.

**Reference**	**Demographics**	**Possible triggers**	**Distribution and clinics**	**Therapy**	**Clinical course, prognosis**
Wojnarowska et al. (2)	UK 25 children 11M/14F 4.5 years	I: 38% infections before onset D: 31% NSAIDS before onset	16 (64%) mucosal involvements; mainly limbs/trunk/head; 15 (60%) perioral lesions; 21 (84%) genitalia/perineum lesions	14 dapsone 8 dapsone + steroids 17 sulphonamides	16 (64%) complete remission 8 (32%) partial remission 1 (4%) lost at follow-up
Aboobaker et al. (17)	African descent 25 children 10M/15F 5 years	I: 4 (16%) infections before onset (2 measles, 1 diarrhea, 1 flu-like)	1 (4%) mucosal involvement; many strings of pearls; face/scalp in all children and generalized rash in the majority of them	8 dapsone 9 dapsone+steroids 3 dapsone+antibiotics 5 more than 3 drugs	5 (20%) complete remission 14 (56%) partial response 6 (24%) lost at follow-up
Singalavanija and Limpongsanurak (16)	Thailand 18 children, 9M/9F, 4.8 years	A: 1 retinoblastoma, 1 neurogenic bladder	No mucosal involvement; 3 (16.6%) strings of pearls	14 dapsone 2 dapsone + steroids 1 dapsone + antibiotics 1 more than 3 drugs	Not reported
Horiguchi et al. (23)	Japan 38 children 5.2 years	None reported	1 (2.6%) mucosal involvement	25 steroids ± dapsone, antihistaminic, topical treatment or self-regression	25 (65.8%) good response 3 (7.9%) partial response
Kenani et al. (24)	Tunisia, 25 children 16M/9F 7.5 years	D: 1 vancomycin course before onset	2 (8%) mucosal involvement; mainly limbs/trunk/head; 20 (80%) strings of pearls	11 dapsone 8 dapsone + steroids 5 antibiotics only	17 (68%) complete remission 8 (32%) partial response
Monia et al. (25)	Tunisia 31 children 13M/18F 5.5 years	A: 1 Jejunal atrophy	4 (12.9%) mucosal involvement; mainly limbs/trunk/head; 10 (32.2%) genitalia/perineum lesions; 12 (38.7%) strings of pearls	8 dapsone 7 dapsone + steroids 4 dapsone + antibiotics 2 antibiotics only	16 (51.6%) complete remission 5 (16.1%) partial remission 10 (32.2%) lost at follow-up
Kong et al. (19)	Singapore 5 children 3M/2F 7.6 years	None reported	No mucosal involvement, mainly limbs/trunk	1 dapsone + steroids 1 steroids 1 colchicine 1 multiple drugs 1 no therapy	3 (60%) complete remission 2 (40%) partial remission
Díaz et al. (26)	Argentina 17 children 10M/7F 3.1 years	A (17.6%): 1 A hepatitis, 1 VATERL, 1 alopecia I (17.6%): 2 flu/varicella vaccine, 1 respiratory disease D (5.9%): 1 antibiotic course before onset	2 (11.8%) mucosal involvement; mainly limbs/trunk/head; 8 (47%) genitalia/perineum involvement	5 dapsone 10 dapsone + steroids; 2 more than 3 drugs	7 (41.2%) complete remission 9 (53%) partial remission 1 (5.9%) lost at follow-up
Genovese et al. (27)	Italy 11 children 8M/3F 5.4 years	A: 1 ulcerative colitis	5 (45.4%) mucosal involvement; mainly limbs/trunk/head; 5 (45.4%) strings of pearls	4 dapsone; 5 dapsone + steroids 1 more than 3 drugs 1 other drug	10 (90.9%) complete remission 1 (9.1%) partial response
Nanda et al. (18)	Kuwait 16 children, 13M/3F 6.4 years	A (50%): 1 PSGN, 1 A thyroiditis, 1 CD, 2 Gilbert, 3 asthma/allergic bronchitis I (37.5%): 4 URTI, 1 viral hepatitis, 1 streptococcus infection	No mucosal involvement; mainly limbs/trunk/head, 10 (62.5%) genitalia/perineum	4 dapsone 4 dapsone + steroids 2 more than 3 drugs 4 cases + IVIG	13 (81.25%) complete remission 2 (12.5%) partial response 1 (6.25%) lost at follow-up

*Complete remission: absence of new and/or established lesions for at least 2 months without or with minimal therapy. Minimal therapy was considered as ≤0.2 mg/kg/day of dapsone and/or 0.1 mg/kg/ day of prednisone (or the equivalent) and/or minimal adjuvant or maintenance therapy. Partial remission: presence of transient (healing within a week) new lesions without or with minimal therapy, as defined above. No response: presence of persistent (not healing within a week) new lesions despite therapy. Relapses were defined as the reappearance of LABD manifestations in patients who showed an at least 4-month duration complete remission*.

*M, male; F, female; UK, United Kingdom; D, drugs; A, autoimmune; I, infections; NSAIDS, non-steroideal antinflammatory drugs; VATERL, vertebral, anal tracheoesophageal, renal and limb defects; PSGN, post-streptococcal glomerulo-nephritis; URTI, upper respiratory tract infections; IVIG, intravenous immunoglobulin; LABD, linear Immunoglobulin A bullous dermatosis*.

**Table 2:** Pediatric cases of LABD: immunohistological findings.

**Reference**	**Immunohistopathological results**		
	**DIF/IDIF**	**IgA/IgG/IgM/C3/others**	**Histology**
Wojnarowska et al. (2)	DIF: 20 linear IgA	4 IgA/IgM, 1 IgA/IgG/IgM/C3	H: 14/25 subepidermal bullae
Aboobaker et al. (17)	DIF: 20 linear IgA	2 IgA/IgG, 4 IgA/C3, 6 IgA/IgM	–
Singalavanija and Limpongsanurak (16)	DIF: 11 linear IgA	1 IgA/C3, 1 IgA/IgM, 4 other combinations	H: 11 subepidermal bullae
Horiguchi et al. (23)	DIF: 35 linear IgA	3 IgA/IgG	–
Kenani et al. (24)	DIF: 16/25 linear IgA	5 IgA/IgM, 3 IgA/C3, 1 IgA/IgM	H: 19/25 subepidermal bullae, 6 microabscesses
Monia et al. (25)	DIF: 12/31 linear IgA;	19/31 combinations IgA/IgG/IgM/C3	H: 23 subepidermal bullae
Kong et al. (19)	DIF: 5/5 linear IgA	–	H: 5/5 subepidermal bullae, 1/5 microabscesses
Díaz et al. (26)	DIF: 17/17 linear IgA	–	–
Genovese et al. (27)	DIF: linear IgA 6/11	5/11 combinations IgA/IgG/IgM/C3	H: 11/11 subepidermal bullae
Nanda et al. (18)	DIF: 16/16 linear IgA	–	–

*DIF, direct immunofluorescence; H, histology*.

### Neonatal Case Series

Some special considerations must be given for the neonatal age (Table 3). As reported above, newborns could be affected during the first days of life; unlike other bullous dermatoses, mothers usually have no clinical manifestations at all (28). The first neonatal case report dates back to 1993 (29), and some other have been published since then (12, 13, 30–38). Some case reports in newborns showed a worse outcome, even a deadly one (36), compared to older children, primarily due to respiratory (13, 36), eye (12), gastrointestinal involvement (31).

**Table 3:** Neonatal cases of LABD.

**Reference**	**Age (days) sex, Country**	**Clinical presentation**	**DIF histology**	**Treatment**	**Clinical course, prognosis**
Hruza et al. (29)	0 day M AA	-Birth: small blisters on face; -At day 4: blisters to genitalia, umbilicus, back, oral mucosa; -At day 6 cough with lung infiltrates; -At day 10 intubation and ventilation required; -At day 30: ECMO	DIF: Linear IgA-IgG-C3 H: Subepidermal bullae with neutrophils and eosinophils	Acyclovir + oxacillin at day 2 Methylprednisolone after day 18 Dapsone added at 6 weeks of life	Due to difficult feeding, a gastrostomy with a Nissen fundoplication was performed; by 5 months of age: poor growth, delayed development, right eye blindness due to scarring, unco-ordinated swallowing Sequelae blindness and uncoordinated swallowing with difficult feeding and poor growth
Gluth et al. (31)	7 days M AA	-Rapidly progressive eruption of blisters on face, tongue, extremities, diaper area, umbilicus; -At day 15 involvement of airways (larynx, trachea) and digestive tract (esophagus)	DIF: linear IgA-IgG-C3	Acyclovir (Tzank initially positive) Dapsone + prednisolone after day 9 IVIG + methylprednisolone at day 24	Tracheostomy at day 15; Relapse after 3 weeks of steroids suspension
Kishida et al. (30)	1 day M Japan	-At day 8 blisters on forehead and trunk, extended to the whole body; -At day 10 intubation for airways obstruction	DIF: linear IgA-C3 (mild staining for IgG and IgM) H: subepidermal bullae with neutrophils and monocytes	Acyclovir + cefazolin Diamonophenylsulfone from day 20 to day 48 At day 48 prednisone due to relapse	Pneumonia; discharged at 5 months of age with mild dysphagia due to pharyngeal scarring and sublingual ranula
Lee (32)	3 days M China	-At day 10 stridor with respiratory failure requiring intubation; -At day 13 small vesicles on face, legs and hands evolved into bullae; -At day 30 swollen larynx at bronchoscopy with vesicles on the left aryepiglottic fold	DIF: linear IgA-IgG-C3 Final diagnosis: bullous pemphigoid and LABD	Prednisolone	All crusted and complete healing within 25 days; extubation at day 38
Akin et al. (33)	6 days M Turkey	-At day 6 localized blisters on neck, cheeks, earlobes and oral cavity, with erythema on the toes, poor weight gain and respiratory distress; at bronchoscopy: bullae in upper respiratory tract and epiglottis. EGDS: bullae on esophagus	DIF: weak linear IgG-C3c at the dermo-epidermal junction. IIF: split skin positive IgA anti-BMZ antibody on the epidermal side	Methylprednisolone for 3 weeks	Still in remission 6 months after treatment. Gastrostomy tube removed 2 months later
Salud and Nicolas (35)	10 days F Philippines	-Mucosal involvement with breathing difficulty requiring intubation; VATERL association	NS	Prednisolone	Transverse loop colostomy was required; pneumonia leading to death
Julapalli et al. (34)	3 days M NS	- At day 3: vesicles on the left neck rapidly spreading to face and back; - At day 5 vesicles spread to scrotum, chest, extremities; few vesicles also in the mouth	DIF: linear IgA (weak IgG-C3) H: subepidermal bullae with neutrophils and eosinophils	Acyclovir, vancomycin, gentamicin desonide ointment at 3 weeks of age	Discharge at 2 weeks of age, no mucosal involvement but recurrent episodes of blisters over neck, buttocks and around the mouth. At 9-month follow-up visit: occasional small blisters primarily on palms and soles, treated with desonide ointment
Romani et al. (12)	3 days M Italy	-At day 3: vesicles on diaper area, neck and face, bilateral mucopurulent conjunctivitis; - At day 4: nasal secretion and oral lesions; next day blisters spread to trunk, limbs, head; - At day 14 respiratory involvement requiring intubation	DIF: linear IgA H: subepidermal bullae with neutrophils and eosinophils	Acyclovir + ampicillin Blepharoconjunctivitis + ulcerative keratitis: dexamethasone + tobramycin + trehalose	Extubating after 3 days; Complete remission at 2 months of age but with severe eye involvement, resulted in right corneal leucoma
Diociaiuti et al. (36)	5 days M Italy	-At day 5 blisters on face and diaper area rapidly spread to body folds and extremities, associated with high fever and respiratory distress requiring intubation; -At 2 months age: extensive oral and corneal erosions, blepharitis	DIF: linear IgA, weak IgG and C3; IgA deposits in trachea and bronchi	Antibiotics, dexamethasone IVIG + methylprednisolone + dapsone	Death at 20 days of life (respiratory failure). Bronchoscopy: tracheobronchial ulcerations and reduced caliber of airways; thorax CT: obliteration of bronchi, atelectasis
Mazurek et al. (37)	0 day M NS	-At birth: bullae and scattered vesicles on cheek, evolved into plaques with yellow crusts, a linear patch of erythema over both eyes with slight yellow crusting; -At day 2: upper back, bilateral conjunctivitis; -At day 3 sublingual vesicles, posterior scleritis	DIF: linear IgA-C3 (weak IgM-IgG) H: subepidermal bullae with neutrophils and eosinophils	Cefotaxine, cloxacillin, acyclovir (5 days) Moxifoxacin for eye involvement, neomycin + dexamethasone + polymixin B; Topical hydrocortisone 1% at day 4 (switched to betamethasone valerate 0.05%)	Discharge at day 11 with the majority of lesions in crusted or resolving phase; at day 17 complete resolution of eye involvement
Giraud et al. (38)	2 days M NS	-At day 2: blisters on limbs, diaper area, perioral region	DIF: linear IgA H: subepidermal bullae with neutrophils and eosinophils	Amoxicillin-clavulanate (maternal fever)	Complete resolution by day 7
Egami et al. (13)	4 days M NS	-At day 4: bullae on neck, buttocks and hands, then extended to oral mucosae; -At day 19 intubation; -At 2 months tracheostomy	DIF: linear deposits of IgA and C3 along BMZ H: Neutrophils, eosinophils, lymphocytes IFF on breast milk: Positive on dermal side	IV fluids steroids, Beta-stimulants, antibiotics	Complete resolution at 6 months

*M, male; F, female; DIF, direct immunofluorescence; IIF, indirect immunofluorescence; H, histology; VATERL, vertebral, anal tracheoesophageal, renal and limb defects; IVIG, intravenous immunoglobulin; ECMO, extracorporeal membrane oxygenation; LABD, linear Immunoglobulin A bullous dermatosis; BMZ, basal membrane zone*.

## Pathology

The typical hallmark of LABD is the deposition along the basement membrane zone (BMZ) of the serum immunoglobulin (Ig) A, which binds to specific antigens located at the dermo-epidermal junction, eventually causing subepidermal blistering (39). It has been demonstrated that in most patients with LABD the main IgA belongs to the subclass A1 (40), although in some patients, IgA2 antibodies have been detected against LABD97 with heterogeneous clinical characteristics in terms of disease onset, duration, gastrointestinal signs and symptoms, or malignancies (40).

The major target antigens are the 120-kDa (LAD-1) and 97-kDa (LABD97) proteins, both parts of ectodomains of BP180 (collagen XVII), and there are cases with autoantibodies against collagen VII, BP230, α6β4 integrin, laminin, and other proteins (39, 41, 42). Target antigens in neonatal LABD are very poorly known. No target antigen has been identified in two patients who underwent immunoblotting studies (12, 33). According to immunoelectron microscopy, LABD shows three patterns of IgA deposition: sub-lamina dense deposition, lamina lucida deposition, and deposition in both layers (43–46). The antigens subset indicates a relationship between Bullous Pemphigoid (BP) and LABD that could be explained by “*the epitope spreading phenomenon*” in which the primary disease, such as ordinary BP, exposes the BMZ to the immune system, which induces autoantibodies against BMZ proteins without pathogenicity. For that reason, BP and LABD may overlap in infantile patients with mucosal involvement (47). LABD histologic and therapeutic findings are similar to those associated with BP and dermatitis herpetiformis (DH), even if LABD and DH are two different diseases in terms of patho-mechanisms and clinical features. LABD can be distinguished from BP and DH by direct immunofluorescence (IF), a demonstration of linear IgA deposits along the basement membrane zone (BMZ). On the contrary, BP has a linear IgG BMZ deposition. Sometimes in LABD, complement, IgG and IgM deposits are described in the BMZ. Indirect IF is frequently negative.

## Pathogenesis

### Genetic

As in DH, a strong association with HLA B8, CW7, and DR3 was reported, without association with gluten-sensitive enteropathy (2, 15, 48, 49). DH is more frequently associated with gluten-sensitive enteropathy and is characterized by granular IgA deposits in the dermal papillae, in direct IF, and the mucosa is not involved. Jejunal atrophy has been described in only one child with LABD (25). In addition, LABD is more likely to happen if these genes are present in a homozygote state (5). HLA B8 and HLADR3 haplotypes were positive in 80% of Tunisian children (24), and in a few studies the positivity of HLA B8 haplotype was associated with a good prognosis (2, 15, 50). Moreover, HLA-DR3-Q2 is considered high risk for type 1 diabetes mellitus (DM) (51), and this genetic pre-disposition, combined with environmental triggers, could probably explain the onset of DM in a child with LABD (52). At last, the role of the TNF2 gene has been described in the duration of the disease, and it was associated with a worse prognosis (53).

### Infections

Most cases of childhood LABD are idiopathic, but infections, medications, skin traumas, and malignancies are potential inducers of LABD. The hypothesis of an infective agent triggering LABD as a result of an immunologic reaction involving IgA has been greatly discussed in the literature. So far, different infectious agents such as Salmonella enteritis (54), non-specific gastrointestinal infection, and Epstein Barr virus infections (55) have been related to LABD in childhood. It is not known if the development of IgA is due to the infection, treatment or both these triggers. Two cases have been reported with an upper respiratory tract infection preceding LABD. In one case, Streptococcus group A was detected in the skin lesions (56). LABD has also been described as following viral hepatitis, post-streptococcal glomerulonephritis, and beta-hemolytic streptococcal throat infections in children (18).

### Drugs

Drug-induced LABD may result from stimulation of the immune system to produce IgA in a susceptible individual. The drugs may disrupt the lamina lucida's antigenicity and act as a hapten, complexing with derma/epidermal proteins and eliciting an autoimmune response. IgA-stimulated neutrophils chemotaxis then results in the formation of neutrophilic microabscesses at the dermo-epidermal junction (14, 57). The interval between drug intake and LABD development ranges from 2 to 28 days and is usually characterized by rapid recovery after stopping the drug. Compared to adults, drug-induced LABD is less frequent in children, with two reports of amoxicillin-clavulanic acid leading to the development of the disease and one case in which remission occurred after cessation of the medication (3, 8). Such reactions to amoxicillin, minocycline, and vibramycin have also been reported in children aged 3, 15, and 17, respectively (58). A retrospective study identified 25 Tunisian children with LABD, and a 6-month-old infant developed LABD 10 days after beginning treatment with vancomycin for bronchopulmonary infection (24). In a report including childhood LABD, childhood cicatricial pemphigoid, and adult LABD, 31% of the patients had taken non-steroidal anti-inflammatory drugs or antibiotics, but no details were provided on which group had received these medications before the eruption. In the same paper, 38% of the children and 26% of the adults had a preceding infection (2). A penicillin-induced LABD has been described in a neonatal case, although recurrence of blistering after drug discontinuation made the diagnosis doubtful (34). Recovery of drug-induced LABD in adults has been reported to take up to several months after discontinuation of an associated agent. Also, a few pediatric cases of drug-induced LABD had a longer duration, such as the one triggered by amoxicillin-clavulanic acid in a 2.5-year-old girl described by Mori et al. (3) in which the recovery took 6 months. Recently, a pediatric case of cephalosporin-induced LABD has been described in a 4-year-old girl after 7 days of antibiotic treatment for a urinary tract infection (5). However, in this case, the concomitant role of the drug and infection in triggering the disease cannot be excluded. In adults, drug-induced LABD cases were described as more severe, resembling toxic epidermal necrolysis (59). In adult case studies of drug-induced LABD, it was found that a minority of patients (20%) have circulating antibodies (60). This contrasts with idiopathic LABD, in which indirect immunofluorescence (IIF) is positive in up to 80% of patients (61). This difference may imply a milder severity as well as a better prognosis for drug-induced LABD. The same conclusion has also been proposed for childhood LABD.

### Underlying Diseases

Association between LABD and underlying diseases has been frequently reported in adults. However, underlying disease has been associated in pediatric patients: ulcerative colitis (62), autoimmune lymphoproliferative syndrome (63), and Crohn's disease (18). Immune activation secondary to the exposition of multiple intestinal epithelial antigens, including BP180, in patients with coexistence of LABD and autoinflammatory bowel disease, has been hypothesized as a possible patho-mechanism eliciting blister formation (64). A case of LABD has been described in a child with pancreatic lipase deficiency (65) and in a child with Gilbert's syndrome (18). Confounding underlying conditions, possible triggering therapies have been reported in pediatric cases of LABD, such as two cases with lymphoblastic leukemia in remission (66) or idiopathic congenital thrombocytopenia and cytomegalovirus infection in children treated with trimethoprim-sulfamethoxazole (7). In one case, the diagnosis of drug-induced LABD was confirmed by a positive cotrimoxazole provocation test (7). To our knowledge, only a case of LABD induced by insect bite in a 22-month-old Chinese girl has been reported in pediatric population (67).

### Vaccinations

Concerning vaccinations as a LABD trigger in children, two cases have been documented after flu/varicella vaccination (26) and two cases after quadrivalent human papillomavirus vaccine (qHPV), respectively in a 14 years old Italian girl (68) and in a 16-year-old Japanese girl (69). It was assumed that, as in rhesus macaques, the qHPV vaccine induces a Th2-skewed immune response with high titers of IgA and IgG1 antibodies, and a high level of IgA could also occur in humans after qHPV vaccine (69). Two cases after SARS-CoV2 vaccination have been reported in adults, one after second dose of AstraZeneca vaccine (70) and one after Pfizer vaccine (71).

### Special Consideration for Neonatal Cases

Today, few cases of neonatal LABD have been described, and the pathomechanism of neonatal LABD is unknown mainly because no data are available for the target antigens. Circulating IgA antibodies against BMZ can be detected, but the origin of IgA deposits in the skin of neonates with LABD remains speculative, as the low level of IgA detected in neonatal cord blood may be produced by the mother or the child (72). Moreover, in all neonatal cases, maternal disease or symptoms were absent during pregnancy, and this fact is striking compared with the mother's frequent involvement in other bullous diseases. It has been postulated that the lower transfer of IgA across the placenta may explain this fact (28). Recently it has been published a case-report of a newborn who developed LABD due to transfer of maternal IgA *via* breast milk. Authors have been able to demonstrate the presence of IgA reactive against BMZ in breast milk, and symptoms disappeared after suspension of breastfeeding (13).

### Clinical Manifestations

**Figure 1:**
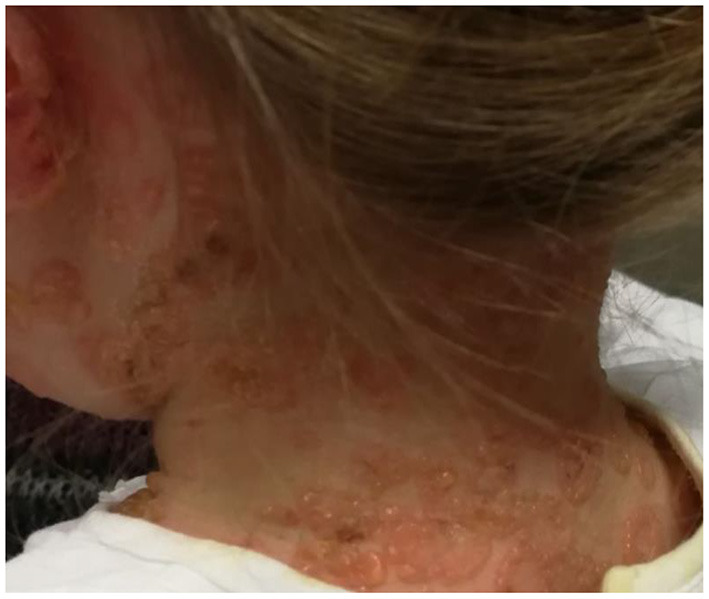
Typical lesions of LABD known as “string of pearls” in a girl evaluated at Meyer's Children Hospital (Florence).

**Figure 2:**
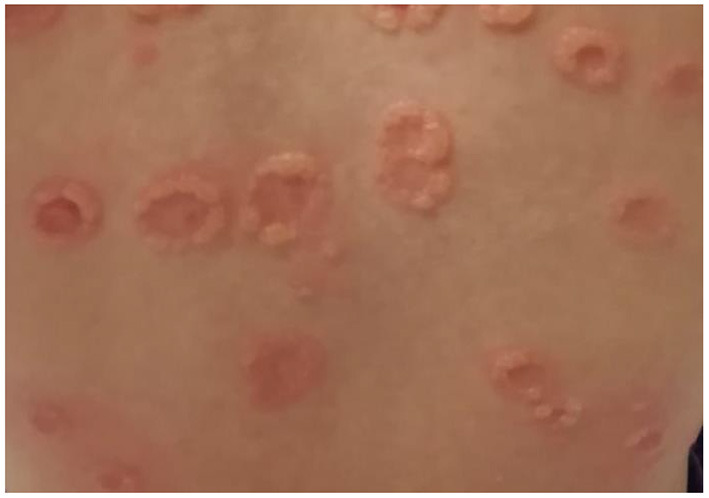
Magnification of typical LABD lesions on the same patient.

The childhood variant involves a sudden eruption of clear or serum-hematic vesicles and blisters on normal or erythematous skin with a typical onset at the abdominal and perioral areas (14, 73, 74). The face (eyes, mouth), hands, and feet may also be affected (75). Skin lesions may be accompanied by pruritus of variable intensity. Commonly there are excoriations and crusted papules, especially in cases of intense itching (76). New blisters develop at the periphery of resolving lesions, resulting in an annular or “rosette-like” pattern (“crown of jewels” or “string of pearls”) (Figures 1, 2, with the permissions of a parents' patient evaluated at Meyer Pediatric Hospital, Florence, Italy).

Lesions may be symmetrically or asymmetrically distributed (77). Data about mucosal involvement are controversial; it has been reported from 8.3% (15) of cases up to 74% (50) of cases. Mucosal involvement, typically of the oral mucosa and conjunctiva, was reported in 64% of the childhood variant cases in Egan et al. study (78) but was rarer (12.9%) in other studies in children (23–25). Mucosal involvement is believed to be less frequent in children than in adults (25, 53), but in a recent study, mucosal involvement was more frequent in children than in adults (27). Even so far, only a few neonatal cases of LABD have been reported in the literature, and mucosal involvement seems to be characteristic of these cases (12, 29–34, 37). Typically, the onset of neonatal LABD is within 10–16 days of life, and in all cases, the skin is affected first. In only one case, lesions were documented at delivery (37). However, skin lesions seem to resolve quickly in neonates. On the other hand, the mucous membrane is involved later but persists longer than skin blisters. Oral lesions consist in erosions and painful ulcerations, desquamative gingivitis, and cicatricial lesions (14). Chronic conjunctivitis can lead to symblepharon, trichiasis, shrinkage of fornices, and even leucoma or blindness (12). Organ involvement, particularly upper airway involvement and less frequent involvement of the gastrointestinal tract, is reported in neonatal cases of LABD except for one (34). Eight patients ended with intubation, and in two cases, tracheostomy was necessary (12, 13, 29–33, 35, 36). Death due to respiratory distress was reported in one patient who also was affected by vertebral, anal, tracheoesophageal, renal and limb defects (VATERL) syndrome, and hypoplastic nasal sinuses (35). Neonatal cases of LABD can show feeding difficulties due to esophageal involvement requiring gastrostomy and ending with permanent scars in a few cases (30, 31).

### Diagnosis

The diagnosis of LABD is established on clinical, histopathological, and immunological data.

Although direct immunofluorescence (DIF) remains the gold standard for diagnosis, the clinician should complete a diagnosis profiling with serum laboratory tests (such a circulating antibodies, complement fractions), serological tests for infective agents, specific exams for autoimmune diseases, complete blood counts, inflammatory and infection biomarkers (such as RCP, ERS).

Histopathological findings are not specific for LABD. Skin biopsy sample usually reveals subepidermal bullae with a predominantly neutrophilic inflammatory infiltrate and sparse eosinophils and lymphocytes. In drug-induced LABD, focal necrotic keratinocytes are more frequent than in idiopathic cases, but the difference is not statistically significant (59). Microabscesses in the dermal papillae can occasionally be seen (25). Laboratory tests are usually normal, although hypereosinophilia is evidenced in some patients (24).

Immunofluorescence studies are suitable, as LABD has many overlapping features with other bullous dermatoses (79). DIF on perilesional skin, shows isolated or predominant continuous IgA deposits at the dermo-epidermal junction (80). Complement fraction 3 (C3c), IgG and/or IgM deposits along the basement membrane zone (BMZ) can also be present at the same time as IgA antibodies (27, 81, 82). There are no specific immunofluorescence patterns that are peculiar for idiopathic rather than drug-induced LABD (59), although, in the latter, linear deposition of C3 at the BMZ is evidenced in almost 1/3 of the cases (83, 84).

A heterogeneous humoral response characterizes LABD. Indirect immunofluorescence (IIF) with salt-split skin was performed to detect circulating autoantibodies anti-BMZ, and its sensitivity ranges between 30% and 50% (26). However, as reported by Antiga et al. (39), circulating IgA are not an exclusive finding in LABD since they could be detected in other subepidermal blistering diseases too (85, 86). Moreover, in LABD, circulating IgG anti-BMZ could be found too (87, 88).

Many cases report associations between LABD and drugs, but there is inadequate evidence of proven causality of them (89). In order to obtain a correct diagnosis, the use of algorithms (e.g., Naranjo score) (59) to evaluate the probability of ADR could be helpful. In particular, a cause–effect relationship is considered when there is definitive evidence that the adverse event occurred after the medication was started, as well as the adverse event diminishes or disappears at any time after stopping the medication.

Provocation tests could be helpful for diagnosis, but they are not usually performed (59) because a severe recurrence (i.e., shorter latency and longer disease course) can occur in rechallenged patients (83).

The primary differential diagnoses in LABD are DH, bullous impetigo, herpes simplex, scabies, arthropod bites, parasitic infections, drug eruptions, dermatitis herpetiformis, and erythema multiforme. Frequently in neonatal cases, the first suspected diagnosis is a bacterial or viral infection (i.e., herpes virus) and most of them received as first treatment intravenous acyclovir plus antibiotics. The differential diagnosis of neonatal blistering includes infectious, autoimmune, and genetic blistering conditions (Table 4).

**Table 4:** Main differential characteristic between neonatal and childhood LABD.

	**Neonatal LABD**	**Childhood LABD**
Clinical characteristics	Mucosal involvement + Upper airways involvement + Gastrointestinal involvement +	Skin involvement + Mucosal involvement ±
Suspected trigger	Infective agents (herpesvirus) Autoimmune diseases Genetic blistering diseases	Idiopathic Drug-induced Infective agents (salmonella; EBV, Streptococcus group A) Insect stings
Pathomechanisms	Unknown Target antigens unknown	IgA against target antigens located at the BMZ such as LAD-1
Prognosis	Bad prognosis	Chronic relapsing course until puberty Benign clinical course

*LABD, linear Immunoglobulin A bullous dermatosis; BMZ, basal membrane zone*.

### Treatment

The primary treatment of LABD is initially dapsone 0.5 mg/kg/ day, which is gradually increased until signs resolve and symptoms are controlled (usually up to 2 mg/kg/day) (9, 10), with a quick response in most of the cases (18, 24, 25, 90). It is effective as monotherapy or in combination with corticosteroids, antibiotics, or colchicine (26). Treatment with dapsone can have adverse effects such as hemolysis in glucose-6-phosphate dehydrogenase (G6PD) deficient patients, methemoglobinemia, bone marrow suppression, and peripheral neuropathy. Therefore, screening for G6PD deficiency must be performed before treatment initiation and levels of methemoglobin, and the blood count with reticulocytes must be monitored regularly (10).

### Corticosteroids

For better disease control, some patients require a low dose of prednisone (0.5 mg/kg/day) in order to suppress blister formation or when there is severe mucosal involvement; on the other hand, the use of corticosteroids should generally be avoided in children because of its long-term side-effects (24).

Sulfapyridine has also been used (91) in patients with G6PD deficiency, or in cases of intolerance to dapsone or sulfapyridine, colchicine (usually 0.6 mg twice daily) can be considered as a possible alternative (9, 92).

### Antibiotics

*Antibiotics*, including erythromycin, oxacillin, flucloxacillin, sulfamethoxypyridazine, and cotrimoxazole, have also been used with variable results (9, 10, 93) even though tetracyclines are not suitable in children <8 years due to the risk of permanent tooth discoloration. Kenani et al. (24) reported favorable outcomes in pediatric patients treated with oxacillin and erythromycin. As these drugs have been proven to be safer than dapsone or steroids, they can be considered a good alternative therapy in LABD. Alajlan et al. (94) described seven pediatric patients with LABD treated with flucloxacillin. They underlined that the precocious beginning of treatment (within 1 month) might be relevant to induce early and long-lasting remission. However, this drug can also determine side effects, such as cholestatic hepatitis, hemolytic anemia, bone marrow suppression, and acute interstitial nephritis (95). Even though these side effects are rare, their monitoring is strongly recommended. According to Mervic et al. (96) miocamycin and a topical corticosteroid (betamethasone dipropionate 0.05% cream, twice daily) can be an effective and safe alternative treatment for LABD patients. It is unclear how these antibiotics work for LABD, although probably through their anti-inflammatory properties.

### Other Drugs

Another drug that has been proven effective in treating LABD is nicotinamide, which has been tested alone (67) or in combination with dapsone (97). The therapeutic function of nicotinamide is to inhibit local factors causing blister formation.

Several adult patients (98, 99) and one pediatric patient (100) were treated with mycophenolate mofetil. Furthermore, patients with LABD have been successfully treated with intravenous immunoglobulins (101).

In some patients with severe skin involvement, in association with systemic therapy, wounded areas can be treated with topic eosin 2%-gentamycin and covered with emollient sterile gauze to prevent bacterial superinfections (6).

Only a few cases of neonatal LABD have been reported in the literature, some of them with significant mucosal involvement (12), ocular lesions, and upper airway and upper aerodigestive tract involvement. In these cases, the use of corticosteroid aerosol therapy and topical ocular corticosteroids were useful.

As LABD is often a benign disease, which in many cases leads to a spontaneous resolution, many authors suggest carefully considering the potential adverse effects of additional treatments by weighing the potential harms and benefits of all drugs. According to a recent study, drug-induced forms of LABD show a more severe clinical evolution than idiopathic LABD (58). Long-term immunosuppression may not be necessary for drug-induced LABD (89) in contrast to idiopathic LABD. In the literature, many studies report a favorable outcome for LABD in childhood. Nanda et al. (18) observed complete remission in 71% of patients after an average treatment period of 1 year.

## Prognosis

Childhood LABD has a chronic relapsing course and resolves before puberty in most cases, often curing without sequelae (10, 14, 22). However, cases with severe morbidity and persisting in adulthood have been reported (8). The disease's mean duration is 14 months (25) and remission has been reported in 64% of children, usually within 2 years. Prompt diagnosis and the right treatment positively affect the prognosis (102). As the disease has a shorter duration and fewer relapses, the clinical course is more benign in children than in adults (73).

In neonatal LABD, respiratory involvement represents the major prognostic factor. The presence of IgA deposits can explain the pathogenesis of respiratory distress through the trachea and bronchi. Indeed, the cylindrical pseudostratified bronchial epithelium forms hemidesmosomes that express BP180, BP230, and α6β4 integrin, leading to mucosal epithelium disruption and fatal bronchial obstruction (36). Few case-reports in neonatal LABD have been described so far, nevertheless most of them showed the severity of such a disease in this age frame. Seven out of twelve (58%) required intubation and only one third showed a complete remission in the follow-up phase. Two patients died and three ended with severe sequelae involving eyes, gastrointestinal or respiratory tracts with scars. The remaining patients still show relapses of the disease.

## Conclusion

Linear immunoglobulin A bullous disease is the most common bullous disease in the pediatric age. It is a disease with unknown exact pathogenesis, and it has been proposed that various stimuli (i.e., infections, drugs, inflammatory diseases, trauma) can activate autoimmune responses through antigen mimicry and epitope spreading. If spontaneous remission occurs, the prognosis is generally favorable. A prompt diagnosis to identify precipitating factors and to timely initiate therapy is crucial to assure a better prognosis.

## Author Contributions

FM and EN conceived the study and supervised it. FM, FS, LL, and AB wrote the manuscript. All the authors performed the research and the selection of the sources, critically revised the manuscript, and accepted the final version of the manuscript.

## Funding

The publication fee was financed by the Italian Society of Pediatric Allergy and Immunology. However, no significant funding source could have influenced the outcomes of this work.

## Conflict of Interest

The authors declare that the research was conducted in the absence of any commercial or financial relationships that could be construed as a potential conflict of interest.

## Publisher's Note

All claims expressed in this article are solely those of the authors and do not necessarily represent those of their affiliated organizations, or those of the publisher, the editors and the reviewers. Any product that may be evaluated in this article, or claim that may be made by its manufacturer, is not guaranteed or endorsed by the publisher.
